# Hormone factors play a favorable role in female head and neck cancer risk

**DOI:** 10.1002/cam4.1136

**Published:** 2017-07-14

**Authors:** Dana Hashim, Samantha Sartori, Carlo La Vecchia, Diego Serraino, Luigino Dal Maso, Eva Negri, Elaine Smith, Fabio Levi, Stefania Boccia, Gabriella Cadoni, Hung N. Luu, Yuan‐Chin Amy Lee, Mia Hashibe, Paolo Boffetta

**Affiliations:** ^1^ The Tisch Cancer Institute Icahn School of Medicine at Mount Sinai New York New York; ^2^ The Zena and Michael A. Weiner Cardiovascular Institute Icahn School of Medicine at Mount Sinai New York New York; ^3^ Department of Biomedical and Clinical Sciences Università degli Studi di Milano Milan Italy; ^4^ Unit of Epidemiology and Biostatistics CRO Aviano National Cancer Institute IRCCS Aviano Italy; ^5^ Department of Biomedical and Clinical Sciences Università degli Studi di Milano Milan Italy; ^6^ Department of Epidemiology University of Iowa College of Public Health Iowa City Iowa; ^7^ Institute of Social and Preventive Medicine (IUMSP) Lausanne University Hospital (CHUV) Lausanne Switzerland; ^8^ Section of Hygiene Public Health Institute Catholic University Rome Italy; ^9^ Head and Neck Surgery Department Institute of Otorhinolaryngology Catholic University Rome Italy; ^10^ Division of Epidemiology Vanderbilt Epidemiology Center Vanderbilt University School of Medicine Nashville Tennessee; ^11^ Department of Epidemiology and Biostatistics College of Public Health University of South Florida Tampa Florida; ^12^ Department of Family and Preventive Medicine and Huntsman Cancer Institute University of Utah School of Medicine Salt Lake City Utah

**Keywords:** Head and neck neoplasms, hormone replacement therapy, mouth neoplasms, reproductive history, women

## Abstract

Due to lower female incidence, estimates of exogenous and endogenous hormonal factors in head and neck cancers (HNCs, comprising cancers of the oral cavity, oropharynx, hypopharynx, and larynx) among women have been inconsistent and unable to account for key HNC risk factors. We pooled data from 11 studies from Europe, North America, and Japan. Analysis included 1572 HNC female cases and 4343 controls. Pooled odds ratios (ORs) estimates and their 95% confidence intervals (CIs) were calculated using multivariate logistic regression models adjusting for tobacco smoking and alcohol drinking. Lower risk was observed in women who used hormone replacement therapy (HRT) (OR = 0.58; 95% CI: 0.34–0.77). Pregnancy (OR = 0.61; 95% CI: 0.42–0.90) and giving birth (OR = 0.59; 95% CI: 0.38–0.90) at <35 years of age were inversely associated with HNCs. An inverse association with HNC was observed with age at start of HRT use (OR = 0.59; 95% CI: 0.39–0.90) for each additional 10 years and with duration of use (OR = 0.87; 95% CI: 0.76–0.99 for every 3 years). Exogenous female hormone use is associated with a nearly twofold risk reduction in female HNCs. The lower female HNC incidence may, in part, be explained by endogenous and exogenous estrogen exposures.

## Introduction

The male‐to‐female incidence ratio of head and neck cancers (HNCs) of the oral cavity, pharynx, oropharynx, hypopharynx, and larynx ranges in the order of 3 to 5 to 1 1. Differences in tobacco smoking, alcohol drinking, and human papilloma virus (HPV) infection do not fully explain this sex incidence ratio 2. Indeed, for laryngeal cancer, the male‐to‐female incidence ratio is the highest among non‐sex‐related neoplasms at 11:1 1. HNC incidence rates are increasing among females 3, 4 although female HNCs are understudied.

The paucity of literature on female HNCs is due to low female case numbers available at each individual institution, low female HNC incidence, and a lack of standardized reproductive or hormone use data collection on HNC patients in general 5. However, estrogen hormone receptors have been shown to be functional 6 in HNC cell lines, expressed depending on hormone ligand stimulation 7, and, recently, correlated with HNC survival status 8. However, the role of hormones in head and neck carcinogenesis in a human model remains unclear. Epidemiological studies support a favorable role of exogenous and endogenous female hormones on HNCs 3, 5, 9. However, these studies included <200 cases and lacked statistical power to concurrently adjust for both tobacco smoking and alcohol drinking confounders.

To clarify the role of hormonal factors in female HNC risk, we pooled primary data from 11 HNC case–control studies in the International Head and Neck Cancer Epidemiology (INHANCE) consortium. This study aimed to evaluate the role of female exogenous and endogenous hormones on female HNC risk and to determine the interaction between female hormones and known risk factors with HNC risk.

## Methods

### Study population

We pooled 1572 female incident HNC cases and 4343 controls from 11 INHANCE studies (http://www.inhance.utah.edu/) 10. Controls were frequency‐matched by age, sex, and race/ethnicity. Controls obtained via random‐digit‐dialing (Iowa and US multicenter studies) or matched‐ based on residence (Milan (2006–2009)) included 1288 (25%) participants (Table S1). Case and control participation rates were greater than 94%, aside from Iowa, US multicenter, and Buffalo studies, which had participation percent rates over 87%, 75%, and 75%, respectively 11.

Incident HNC cases were defined by the following codes of the World Health Organization (WHO) International Classification of Disease (ICD‐O2 Version 2 12, ICD‐9 13, or ICD‐10 14) and included: oral cavity (*n* = 558), (C00.3‐C00.9, C02.0‐C02.3, C03.0, C03.1, C03.9, C04.0, C04.1, C04.8‐ C05.0, C06.0‐C06.2, C06.8, C06.9); oropharynx (*n* = 339), (C01.9, C02.4, C05.1, C05.2, C09.0, C09.1, C09.8, C09.9, C10.0‐C10.4, C10.8, C10.9); hypopharynx (*n* = 99) (C12.9, C13.0‐13.2, C13.8, C13.9); and larynx (*n* = 212) (C32.0‐C32.3, C32.8, C32.9), as well as pharynx not otherwise specified (*n* = 302) and overlapping HN sites (*n* = 62). Due to the specific HPV‐driven etiology of oropharyngeal cancers 15, they were analyzed separately.

### Exposure variables

Data were collected by trained interviewers using structured questionnaires including menstrual and reproductive information, lifelong use of oral contraceptives (OC) and hormone replacement therapy (HRT), age at menopause and menarche, socio‐demographics, and smoking and drinking habits. HRT therapy was defined as hormone therapy for menopausal symptoms. Written informed consent was obtained from all study subjects; each institutional review board approved investigations.

Questionnaire data were collected from each study to assess question wording and data comparability, for data harmonization. Reliability for HRT therapy and OC use were over 90% and 93%, respectively; for parity and age at menopause, 94% and 66%, respectively 16, 17, 18. Study subjects had a mean age±standard deviation(SD) of 59.0 ± 0.17 years, within the upper range of the world menopausal age average 19. Categorical age cut‐offs at menopause and menarche were chosen based on the pooled data median. First birth and pregnancy 35+ years was chosen based on the clinical definition of advanced maternal age, hormonal changes, and cancer risk 20, 21. Given the well‐established relationship between body mass index (BMI) and female hormones, we also analyzed the association between BMI with HNCs 22, 23. In most studies (both Milan studies, Aviano, Italy Multicenter, Switzerland, and US Multicenter) BMI was ascertained at enrollment, while in others (Buffalo, Iowa, Rome, and both Japan studies) BMI was ascertained 2–5 years earlier. The Pearson correlations were over 0.88, and BMI odds ratios (ORs) for HNC were similar between all studies. Alcohol consumption was calculated in standardized drinks/day 24.

### Statistical analyses

Associations between hormone factors and HNC were assessed by OR and corresponding 95% confidence intervals (CIs) using multivariate logistic regression models. To determine summary associations, study‐specific estimates were included in a multivariate two‐stage random‐effects logistic regression model 25, allowing for study heterogeneity. Variables with the largest number of data by study are displayed using both random‐effects models. Pooled odds ratios were also estimated with a fixed‐effects logistic regression model for each hormone variable, adjusting for age, education, study, BMI, tobacco smoking, and alcohol, as described previously 3, 26, 27.

For stratified analyses, separate multivariable models were run for never and former/current smokers to minimize the possible confounding of former smokers. The same method was applied to alcohol drinking. BMI categories were separated as: underweight‐ <18.5 kg/m^2^, normal‐18.5–24.9 kg/m^2^, overweight‐25–29.9 kg/m^2^, and obese‐ ≥30 kg/m^2^. We also included interaction terms to determine modifying effects between hormone variables and smoking and drinking. A Student's *t*‐test was used to determine whether means for months of exogenous hormone differed between cases and controls. All analyses were performed using Stata version 14.0 (StataCorp., College Station, Texas) and *P* < 0.05 was considered significant.

## Results

The distributions of cases and controls by selected characteristics are reported in Table S2. A total of 546 HNC cases (36.6%) were nondrinkers versus 1882 (47.2%) controls who were nondrinkers. Nonsmokers included 506 (33.5%) cases and 2536 (63.7%) controls. A high school education or more was inversely associated with HNCs (OR = 0.77; 95% CI: 0.65–0.92) with a dose–response effect (*P*_*trend*_ based on five categories<0.001). This association remained after adjustment for hormonal factors; effect modification with hormone variables was *P *=* *0.76. Mean BMI for cases was 23.1 kg/m^2^ ± 0.12 standard deviation (SD) versus controls 24.5 kg/m^2^ ± 0.13 SD; *P *<* *0.001. No association was observed for HNCs by BMI category (Table S3); effect modification for BMI with all hormone variables were all *P *>* *0.05; data not shown.

### Exogenous hormone use

Inverse associations were observed across all HNC and cancer sites for both HRT and OC use (Table 1). Respective ORs and 95% CIs between ever using HRT and HNC, oral cavity cancer, and oropharyngeal cancer were 0.58 (0.34–0.77), 0.56 (0.32–0.97), and 0.49 (0.31–0.77), respectively. For OC use, a dose–response relationship with age was observed, with inverse associations for those who started at 31+ years old (OR = 0.37, 95% CI: 0.22–0.63; *P*_*trend *_< 0.001). When age at first OC use was treated as a continuous variable, a similar inverse association was observed: OR = 0.61; 95% CI: 0.47–0.82 for all HNCs and oropharyngeal cancer (OR = 0.60; 95% CI: 0.39–0.92), but not oral cavity cancer (OR = 0.67; 95% CI: 0.42–1.07).

**Table 1:** Associations between exogenous and endogenous hormone‐related factors with the risk of head and neck cancer and by subsite in INHANCE Consortium

	Overall Head and Neck Cancers		Oral Cavity		Oropharynx	
	Cases/Controls	OR (95% CI)	Cases/Controls	OR (95% CI)	Cases/Controls	OR (95% CI)
*Exogenous Hormone factors*						
Ever used HRT^a^						
Never	370/791	1.0 (Ref.)	139/791	1.0 (Ref.)	134/791	1.0 (Ref.)
Ever	626/1,351	0.58 (0.34–0.77)	266/1,351	**0.56 (0.32**–**0.97)**	325/1,351	0.49 (0.31–0.77)
Age at first HRT use (years)						
Never	122/521	1.0 (ref.)	41/521	1.0 (ref.)	60/521	1.0 (ref.)
<42	51/98	1.59 (0.47–5.35)	21/98	1.05 (0.10–10.52)	29/98	0.82 (0.08–8.77)
42+	42/112	0.93 (0.27–3.18)	16/112	0.64 (0.06–7.34)	25/112	2.17 (0.23–20.19)
*P*_*trend*_		0.32		0.34		0.18
Ever used OC^b^						
Never	349/1,373	1.0 (Ref.)	110/1,373	1.0 (Ref.)	150/1,373	1.0 (Ref.)
Ever	88/335	0.59 (0.40–0.86)	31/335	0.64 (0.36–1.17)	33/335	0.55 (0.29–1.07)
Age at first OC use (years)						
Never	348/1,372	1.0 (Ref.)	109/1,372	1.0 (Ref.)	150/1,372	1.0 (Ref.)
<31	61/182	0.83 (0.52–1.34)	18/182	0.73 (0.35–1.54)	25/182	0.86 (0.38–1.95)
31+	30/180	0.37 (0.22–0.63)	14/180	0.55 (0.26–1.18)	10/180	0.31 (0.12–0.79)
*P*_*trend*_		<0.001		0.12		0.02
*Endogenous hormone factors*						
Ever given birth						
No	161/360	1.0 (Ref.)	60/360	1.0 (Ref.)	74/360	1.0 (Ref.)
Yes	400/1612	0.70 (0.52, 0.95)	136/1,612	0.74 (0.47–1.16)	152/1612	0.61 (0.36–1.02)
Age at first pregnancy (years)						
Never	119/248	1.0 (Ref.)	47/248	1.0 (Ref.)	54/248	1.0 (Ref.)
<35	555/1980	**0.61 (0.42**–**0.90)**	187/1,980	0.55 (0.30–0.98)	261/1,980	0.48 (0.25–0.93)
35+	17/47	0.82 (0.39–1.71)	5/47	0.65 (0.21–2.08)	8/47	0.43 (0.09–2.13)
*P*_*trend*_		0.09		0.11		0.04
Age at first birth (years)						
Never	112/232	1.0 (Ref.)	42/232	1.0 (Ref.)	53/232	1.0 (Ref.)
<35	544/1,925	0.59 (0.38–0.90)	182/1,925	0.44 (0.22–0.90)	147/1925	0.48 (0.24–0.95)
35+	17/47	0.78 (0.37–1.65)	5/47	0.50 (0.14–1.61)	8/47	0.39 (0.08–1.94)
*P*_*trend*_		**0.03**		0.03		0.04
Breastfeeding						
No	391/1,197	1.0 (Ref.)	127/1197	1.0 (Ref.)	209/1,197	1.0 (Ref.)
Yes	125/361	0.97 (0.67–1.43)	44/361	1.02 (0.55–1.88)	54/361	0.98 (0.50–1.96)
Menarche age						
14 years+	322/1024	1.0 (Ref.)	110/1024	1.0 (Ref.)	88/1024	1.0 (Ref.)
<14 years	359/1307	0.89 (0.73–1.10)	111/1307	0.81 (0.58–1.12)	95/1307	0.74 (0.50–1.11)
Menopause cause						
Pre‐and peri menopause	115/510	1.0 (Ref.)	41/510	1.0 (Ref.)	56/510	1.0 (Ref.)
Natural	513/1746	1.79 (1.16–2.77)	184/1,746	1.44 (0.72–2.87)	231/1746	1.98 (0.92–4.28)
Surgical	124/423	1.62 (1.02–2.56)	46/423	1.37 (0.67–2.81)	52/423	1.89 (0.92–3.88)
Menopause age						
Never	70/416	1.0 (Ref.)	22/416	1.0 (Ref.)	41/416	1.0 (Ref.)
<52 years	476/1494	1.69 (1.06–2.71)	168/1494	0.94 (0.44–2.01)	212/1,494	2.52 (1.02–6.27)
52+ years	172/701	1.54 (0.93–2.57)	63/701	0.86 (0.38–1.92)	78/701	1.88 (0.68–5.24)
*P*_*trend*_		0.33		0.56		0.95

Models adjusted for study (and center for multicenter studies), age (<40, 40–44, 45–49, 50–54, 55–59, 60–64, 65–69, 70–74, 75 +  years), education level (≤high school, > high school), amount of alcohol drinking (nondrinker, 0.1–0.9, 1.0–2.9, 3.0–4.9, and 5.0+ drinks/day), body mass index (normal weight, underweight, overweight, and obese), and cumulative tobacco smoking (never smoker, smoked 0–10.0, 10.1–20.0, 20.1–30.0, 30.1–40.0, 40.1–50.0, 50.0+ pack‐years).

a HRT, hormone replacement therapy.

b OC, oral contraceptives; OR: Odds ratios.

Among women who used HRT, an inverse association with HNC was OR = 0.59 (95% CI: 0.39–0.90) for each additional 10 years of age at which HRT was started. For every additional 3 years that HRT use continued, HNC risk decreased by 13% (OR = 0.87; 95% CI: 0.76–0.99). Only seven cases and 26 controls had ever used both HRT and OC. There was no significant difference between months of HRT use (25.6 ± 60.3 SD vs. 23.5 ± 63.6 SD; *P *=* *0.61) or months of OC use (10.4 ± 32.5 SD vs. 8.2 ± 28.5 SD; *P *=* *0.16) between cases and controls, respectively.

### Endogenous hormones: reproductive and menstrual variables

Giving birth was inversely associated with all HNCs. An inverse relationship was observed for age at first pregnancy and age at first birth <35 years versus never having been pregnant or given birth for all HNCs, oral cancers, and oropharyngeal cancers. The number of births was not significantly associated with HNCs [Respective ORs and 95% CIs for HNC: 0.94 (0.88–1.01), oral cavity: 0.93 (0.83–1.04), and pharynx: 0.92 (0.83–1.02)] (data not shown). There were no appreciable associations between having had a miscarriage or abortion with HNCs, or with subsites (data not shown).

Menopause at <52 years old was associated with higher risk of all HNCs. Menopause due to both natural and surgical causes was associated with higher risk of HNCs compared to pre‐ and peri‐menopausal women. However, when menopause type was adjusted for HRT use, associations attenuated for both naturally and surgically induced menopause [(Natural menopause: OR = 1.46; 95% CI: 0.74–2.85; *n* = 98 HRT use/*n* = 145 no HRT use) and Surgical menopause: OR = 1.17; 95% CI: 0.58–2.37; (*n* = 25 HRT use/*n* = 45 no HRT use)]. No association was observed between age at menarche with all HNCs or subsites, both in continuous scale (data not shown) and categorical scale (<14 vs. 14+ years of age). Similarly, menstrual cycle duration (OR = 1.04; 95% CI: 0.94–1.14) and menstrual period duration (OR = 0.96; 95% CI: 0.87–1.05) were not associated with HNCs (data not shown).

**Figure 1:**
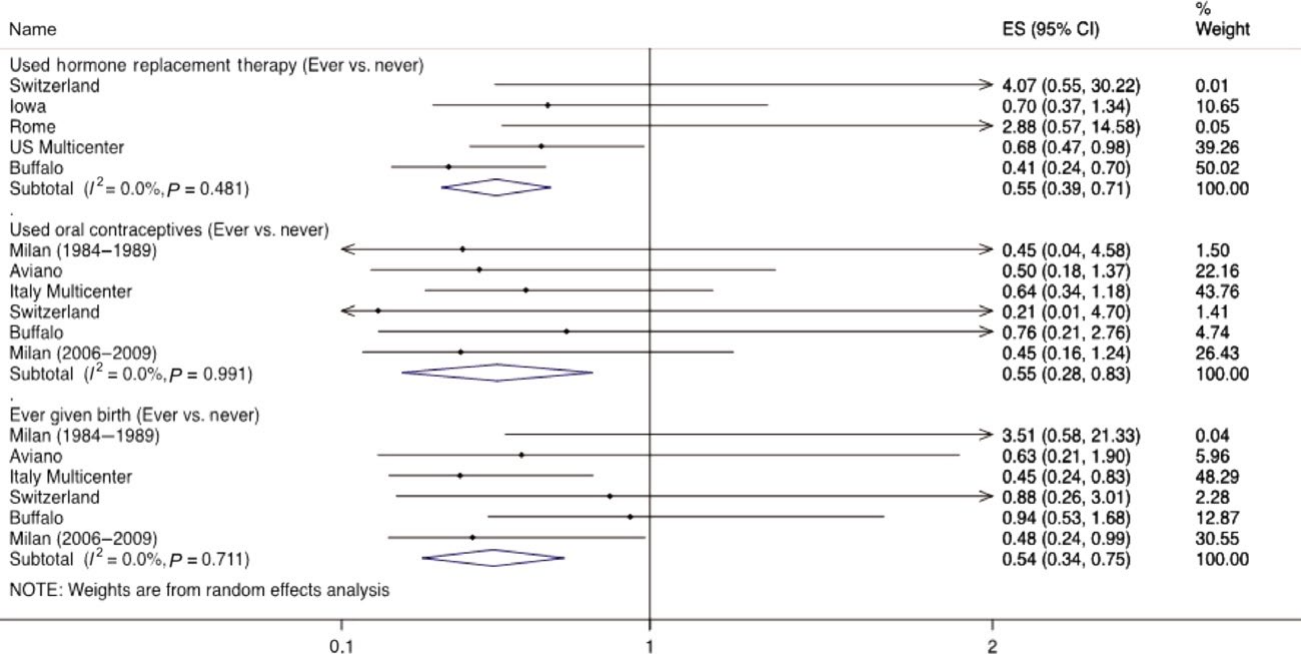
Association between the use of HRT, oral contraceptives, and ever given birth with the risk of head and neck cancer by study in INHANCE Consortium.

Between‐study heterogeneity was not detected. Inverse associations were observed between ever using HRT and all HNCs (Fig.**** 1) as well as oral cavity and oropharyngeal cancers in random‐effects models [Respective pooled ORs and 95% CIs: 0.58 (0.31–0.84); 0.49 (0.29–0.75)]. Similar to fixed‐effects models, ever OC use was inversely associated with overall HNCs [Respective pooled ORs and 95% CIs: 0.55 (0.28–0.83)] but not for oropharyngeal cancers: 0.46 (0.06–0.98). Ever giving birth was inversely associated with all HNCs as well as both oral cavity and oropharynx cancer subsites [Respective pooled ORs and 95% CIs: 0.54 (0.34–0.75); 0.25 (0.10–0.41); 0.54 (0.21–0.82)].

### HNC risk and hormones, by smoking and drinking

#### HRT use

Ever using HRT remained inversely associated with all HNCs, oral cavity, and oropharyngeal cancers in ever‐smokers only (Table 2). An interaction between smoking status and HRT was observed for oropharyngeal cancer. Interactions for HRT and alcohol drinking were observed for HNCs and oral cavity cancers (*P*_*interaction *_= 0.01 and 0.002, respectively).

**Table 2:** Risk of head and neck cancer overall and for subsites in tobacco smokers and alcohol drinkers within strata of endogenous and exogenous hormones variables, INHANCE Consortium

	Overall head and neck				Oral cavity				Oropharynx			
	Cases (E/E–)	Controls (E/E–)	OR (95% CI)	*P*_*interact*_	Cases (E/E–)	Controls (E/E–)	OR (95% CI)	*P*_*interact*_	Cases (E/E–)	Controls (E/E–)	OR (95% CI)	*P*_*interact*_
Ever used HRT (ever vs. never)												
Alcohol drinking status				0.01				0.002				0.75
Never	133/191	415/638	0.50 (0.25–1.00)		44/95	415/638	0.44 (0.15–1.26)		10/45	415/638	0.47 (0.15–1.48)	
Ever	219/427	330/685	0.61 (0.36–1.05)		92/167	330/685	0.70 (0.36–1.37)		42/127	330/685	0.48 (0.29–0.80)	
Tobacco smoking status				0.06				0.31				0.02
Never	98/199	492/864	0.49 (0.23–1.04)		41/104	492/864	1.04 (0.63–1.73)		11/37	492/864	0.70 (0.25–2.02)	
Ever	267/426	283/479	0.50 (0.31–0.82)		97/161	283/479	0.58 (0.39–0.85)		41/137	283/479	0.45 (0.27–0.75)	
Ever used OC (ever vs. never)												
Alcohol drinking status				0.02				0.61				0.14
Never	21/75	97/532	0.74 (0.34–1.61)		6/30	97/532	0.53 (0.15–1.90)		4/20	97/532	0.35 (0.07–1.88)	
Ever	63/273	226/798	0.53 (0.34–0.83)		23/76	226/798	0.65 (0.32–1.32)		18/98	226/798	0.54 (0.26–1.10)	
Tobacco smoking status				0.21				0.60				0.01
Never	24/98	184/991	0.82 (0.46–1.47)		8/41	184/991	0.56 (0.22–1.45)		8/21	184/991	1.20 (0.43–3.38)	
Ever	63/249	147/378	0.63 (0.39–1.01)		23/68	147/378	0.83 (0.37–1.82)		13/62	147/378	0.39 (0.17–0.91)	
Ever given birth (ever vs. never)												
Alcohol drinking status				0.28				0.45				0.08
Never	103/41	565/160	0.89 (0.61–1.30)		42/15	565/160	0.79 (0.44–1.40)		22/9	565/160	0.85 (0.29–2.50)	
Ever	287/117	990/191	0.50 (0.29–0.84)		92/45	990/191	0.78 (0.35–1.72)		67/33	990/191	0.68 (0.36–1.25)	
Tobacco smoking status				<0.001				0.12				0.06
Never	104/42	1074/217	0.80 (0.50–1.27)		42/15	1074/217	0.81 (0.42–1.56)		30/5	1,074/217	1.39 (0.46–4.20)	
Ever	278/111	520/139	0.68 (0.46–1.00)		89/43	520/139	0.77 (0.42–1.43)		59/39	520/139	0.46 (0.25–0.88)	

Models adjusted for study (and center for multicenter studies), age (<40, 40–44, 45–49, 50–54, 55–59, 60–64, 65–69, 70–74, 75+ years), education level (≤high school, > high school), amount of alcohol drinking (nondrinker, 0.1–0.9, 1.0–2.9, 3.0–4.9, and 5.0+ drinks/day), body mass index (normal weight, underweight, overweight, and obese), and cumulative tobacco smoking (never smoker, smoked 0–10.0, 10.1–20.0, 20.1–30.0, 30.1–40.0, 40.1–50.0, 50.0+ pack‐years).

E, exposed; E–, unexposed; HRT, hormone replacement therapy; OC, oral contraceptives; OR, Odds ratios.

#### OC use

The association between oropharyngeal cancer and ever OC use was inversely associated for ever‐smokers, but not observed for never‐smokers. No interaction between smoking and OC use was observed for HNC overall. However, an inverse association was observed between OC use and all HNCs in stratified analyses for ever‐drinkers, with significant interaction (*P*_*interaction*_=0.02), but not for never‐drinkers.

#### Ever given birth

No interactions were observed between giving birth and HNCs when stratified for alcohol drinking. An interaction was observed for HNCs and smoking (*P*_*interaction  = *_0.02). The risk of oropharyngeal cancer was lower for ever‐smokers who had given birth than for never‐smokers. Interactions between hormone variables and BMI for HNC risk were: *P *=* *0.15 for HRT use, *P *=* *0.50 for OC use, and *P *=* *0.78 for ever given birth.

## Discussion

This study accomplished an adequately powered investigation of major exogenous and endogenous hormone‐related variables on female total HNCs as well as oral cavity cancers, and oropharyngeal cancer subsite risk by pooling primary data from multiple studies. We confirmed an inverse relationship between multiple estrogen‐progesterone hormone factors and HNC risk among females, an understudied population. Associations were favorable for high‐estrogen endogenous states, specifically earlier first pregnancy and earlier menarche, and inconsistent for age at menopause which supports risk susceptibility at younger age of exposure. The relationship between hormones factors and HNCs appears to be modified by alcohol drinking and smoking status, consistent with known biological mechanisms of these cancer risk factors on female hormones. Considering our stratification for tobacco and alcohol exposure still resulted in inverse associations for hormone variables among those with high cancer risk, we interpret this to mean that the decrease in female HNC risk could not be solely explained by tobacco or alcohol alone.

Some studies on female HNCs suggested a favorable effect of some hormone factors 28, while others found null associations 28. Accumulated, the evidence supports a mixed causal effect of both sex (biological) and gender (psycho‐sociological) differences on HNC 29, 30 with a protective relationship with younger exposure age.

Biologically higher natural estrogen levels among women have been proposed as an explanation for lower risk of HNCs compared to men. As ~90% of HNCs are squamous cell carcinomas, this hypothesis is analogous with findings among other upper‐thoracic squamous cell carcinomas in women demonstrating a favorable associations of female combination hormones for cancers of the oral cavity and pharynx 3, and esophagus 31, including a prospective study 32.Associations for hormone use with esophageal squamous cell carcinoma were stronger than for adenocarcinoma 31, 32. For HNCs specifically, experimental in vitro and in vivo studies support a plausible biological mechanism for hormone ligand action. Lukits and colleagues 6 demonstrated evidence for estrogen and progesterone receptors in 67 of human HNC surgical samples. Colella et al.^33^ found that hormone expression patterns differ between malignant versus nonmalignant oral mucosa tissue. The beta estrogen receptor (ERβ) in particular, functions as a tumor suppressor by inhibiting proliferation and driving cellular differentiation 34 and ERβ positivity has been correlated with improved survival of oropharyngeal cancer patients 8. Moreover, the selective estrogen receptor modulator, tamoxifen, has been shown to arrest G1 in HNC cell lines, sensitizing cancer cells to chemotherapy‐induced apoptosis 35, 36. These findings indicate that specific biological mechanisms exist by which hormone levels can influence HNC phenotypes when receptors are activated.

A second explanation for the inverse relationship between estrogenic hormones on female HNCs is the influence of estrogen on liver metabolism. *CYP1B1*, part of the Cytochrome P450 enzyme superfamily, plays a key role in sex hormone metabolism and homeostasis. Shatalova et al.^37^ found estrogen exposure of premalignant HNC cells reduced *CYP1B1* gene expression. Yoo et al. 38 examined estrogen metabolites in 50:50 HNC case–controls and found higher abnormal estrogen metabolism expression among cases (OR = 15.6;95% CI: 1.1–212.5). The stratified hormone analyses for our study showed a stronger inverse relationship among ever‐drinkers. Given that consistent alcohol drinkers have altered liver metabolism and higher estrogen levels, it is plausible that the combination of exogenous estrogen level exposure and alcohol may work in tandem to decrease HNC risk estimates and may explain P values < 0.05 for interaction as well as an inverse association for female ever‐drinkers (OR = 0.53[95% CI: 0.34–0.83]. This pattern was confirmed for our study when we restricted analyses to heavy drinkers (≥3.0 drinks per day) (OR for HRT use = 0.34; 95% CI: 0.02–6.61 and OR for OC use = 0.36;95% CI: 0.12–1.13). Although these CIs include 1, an inverse pattern is prevalent for heavy drinkers with a greater inverse association than among ever‐drinkers. It must also be noted that these analyses were restricted to 169 and HNC cases and 222 controls due to the low prevalence of female heavy drinkers.

Considering that women have considerably lower alcohol consumption and tobacco use than men (psycho‐sociological gender‐related differences), it is not surprising that female HNC incidence is also lower. Modification of tobacco smoking/alcohol drinking by female hormones on HNC that were observed in our study may partly explain the lower HNC incidence rate among women in the general population. This would explain psycho‐sociological factors associated with fewer female HNCs.

Tobacco smoking interacts with female hormones, increasing hepatic metabolism and estrogen catabolism 39, 40, 41. Increased estrogenic bioavailability augments the risk of estrogenic side effects such as deep vein thrombosis and female patients who are smokers are routinely cautioned against OC use. Partly due to this medical contraindication, there were too few individuals who both used OC and smoked in our study to power an accurate association estimate.

Although stratified analyses by BMI were hindered by low numbers for most BMI categories, the patterns of a J‐shaped association curve for BMI with HNC are consistent with hormonally associated risk changes found in studies for other cancers 42, 43, 44.The dose–response decrease in HNC risk with longer HRT use duration offers further evidence for a real relationship. Furthermore, earlier age at pregnancy and/or birth were also favorably associated with HNC risk than never being pregnant or having given birth. This also supports the notion that there is a specific age range of hormone exposure that benefits women at younger reproductive age, similar to observations found for breast cancer 6, 45.

Due to the predominant role of HPV, causing approximately 70% of oropharyngeal cancers 15, oropharyngeal cancer must be uniquely considered in light of female hormones. At any given time, approximately 4–7% of individuals are expected to have oral or oropharyngeal HPV infection 46, 47. In half of these individuals, these infections are cleared by the immune system 48. It is already been observed that men are more likely to have an HPV infection than women and that sexual behavior differences could only explain 16% of this prevalence difference 46. Women also have a higher preponderance for autoimmune diseases such as Hashimoto thyroiditis and multiple sclerosis than men and have a stronger allograph rejection response than men, indicating that certain diseases are indeed mediated by female sex hormones 49, 50. This is supported by the observation that sex hormones have direct effects mediated via hormone receptors in immune cells, act through their actions on epithelial cells and stromal fibroblast secretion of growth factors 51, and has been observed to alter epithelial cell proliferation in the female reproductive tract 50. In an epidemiological study among postmenopausal women, who have lower physiological estrogen states, reduced immunologic fitness was observed to account for 12% of HPV infections 52. However, the mechanism for how the stronger immune response conferred by female sex hormones affects oropharyngeal cancers in response to HPV infection has not been examined, to the best of our knowledge, and warrants further study.

As with all case–control studies, recall bias is a limitation. However, we used incident cases, which is distinct from cases identified before data on risk factors were collected, making recall bias less likely. Many study centers were hospital‐based, leading to selection bias suspicion of controls with hormone use‐related conditions. However, the majority of hospital controls were unhealthy, hospitalized due to other illnesses unrelated to hormone use, rather than cancers. Another limitation of hospital‐based studies is lack of external validity, whereby the hospital population differs from the general population. However, the fact that we also found inverse associations for hormone variables with HNCs across the population‐based studies of Iowa, Milan (2006–2009), and US multicenter contradicts the possibility of hospital‐based selection bias. Further, the magnitudes of the inverse associations that we have observed in our study are not dissimilar to previous prospective studies on female HNC and hormone factors 53, 54.

Our study is strengthened by a high number of female incident HNC cases, enabling us to evaluate the relationships between female hormones and HNC, and relationships stratified by tobacco smoking and alcohol drinking. The consistency of findings for high‐estrogen states among both endogenous and exogenous hormone factors supports a true inverse association between estrogen and HNC, but this association may be stronger at earlier age of exposure 55. The HRT users in our study were taking HRT for menopausal symptoms, which consisted of both estrogen and progesterone combined rather than unopposed estrogen. This warrants further experimental studies to understanding the mechanisms behind which female hormones act on head and neck cells to prevent or reduce cancer risk.

In conclusion, this study supports a favorable association of female hormones with HNC. It also suggests that the lower incidence of female HNCs may be due to a combination of exogenous and endogenous hormone exposure and different smoking and drinking risk exposures.

## Conflict of Interest

All authors claim no conflict of interest.

## Supporting information


**Table S1.** Selected characteristics of studies included in the pooled analysis of hormone and reproductive related variables and head and neck cancer, INHANCE Consortium.
**Table S2.** Demographic characteristics of head and neck cancer cases and controls, INHANCE Consortium.
**Table S3.** Associations between hormone and reproductive variables and all head and neck cancers in strata of body mass index (BMI, kg/m^2^), INHANCE Consortium.
