# Supplementary material for: Hormone factors play a favorable role in female head and neck cancer risk
Source: Cancer Med. 2017 Jul 14;6(8):1998–2007. doi: 10.1002/cam4.1136 (PMC5548879; doi:10.1002/cam4.1136)
Supplement: Supplementary file 1 — Table S1. Selected characteristics of studies included in the pooled analysis of hormone and reproductive related variables and head and neck cancer, INHANCE Consortium. Table S2. Demographic characteristics of head and neck cancer cases and controls, INHANCE Consortium. Table S3. Associations between hormone and reproductive variables and all head and neck cancers in strata of body mass index (BMI, kg/m2), INHANCE Consortium. [file CAM4-6-1998-s001.docx]

**Table S1. Selected characteristics of studies included in the pooled analysis of hormone and reproductive related variables and head and neck cancer, INHANCE Consortium**

|  |  | Endogenous hormone variables | | | | Exogenous hormone variables | | | | | | |  |  |
| --- | --- | --- | --- | --- | --- | --- | --- | --- | --- | --- | --- | --- | --- | --- |
| Study | **Years** | **HRT^a^ use** | **Age at HRT use** | **OC^b^ use** | **Age at OC use** | **Ever given birth** | **Age at first preg-nancy** | **Ever breastfed** | **BMI^c^** | **Age at menarche** | **Age at meno-pause** | **Reason for meno-**  **pause** | **N cases/**  **controls** | **Control source** |
| Milan 1 | 1984-1989 |  | x | x | x | x | x |  | x | x | x | x | 40/400 | Hosp (un) ^d^ |
| Aviano | 1987-1992 |  |  | x | x | x | x |  | x | x | x | x | 51/180 | Hosp (un) |
| Italy multi | 1990-2005 |  |  | x | x | x | x | x | x | x | x | x | 191/991 | Hosp (un) |
| Switzerland | 1991-1997 | x | x | x | x | x | x | x | x | x | x | x | 88/266 | Hosp (un) |
| Iowa | 1993-2006 | x |  |  |  |  |  |  |  |  |  |  | 197/308 | RDD^d^ |
| Rome | 2002-2007 | x |  |  |  |  |  |  |  |  |  |  | 70/167 | Hosp (un) |
| US multi | 1983-1984 | x | x |  |  |  |  |  | x |  |  |  | 352/431 | RDD |
| Japan 2 | 2001-2005 | x |  |  |  |  |  |  | x |  | x | x | 126/621 | Hosp |
| Japan 1 | 1988-2000 |  |  |  |  |  | x | x | x | x | x | x | 194/444 | Hosp |
| Buffalo | 1982-1998 | x |  | x | x | x | x | x | x | x | x | x | 193/386 | Hosp |
| Milan 2 | 2006-2009 | x | x | x | x | x | x | x | x | x | x | x | 68/266 | Resid |
| Total |  |  |  |  |  |  |  |  |  |  |  |  | 1,572/4,343 |  |

^a^HRT: Hormone replacement therapy

^b^OC: Oral contraceptives

^c^BMI: Body mass index (kg/m^2^)

^d^RDD, random digit dialing; un: “unhealthy” controls with a non-HNC malignancy

Hosp: hospital; Resid: residence

**Table S2. Demographic characteristics of head and neck cancer cases and controls, INHANCE Consortium**

| Characteristics | Cases (1510) | | Controls (3984) | | Total (N) |
| --- | --- | --- | --- | --- | --- |
|  | **n** | **%** | **n** | **%** |  |
| Age (years) |  |  |  |  |  |
| <40 | 89 | 5.89 | 326 | 8.18 | 415 |
| 40-44 | 54 | 3.58 | 229 | 5.75 | 283 |
| 45-49 | 129 | 8.54 | 370 | 9.29 | 499 |
| 50-54 | 164 | 10.86 | 467 | 11.72 | 631 |
| 55-59 | 232 | 15.36 | 596 | 14.96 | 828 |
| 60-64 | 246 | 16.29 | 571 | 14.33 | 817 |
| 65-69 | 251 | 16.62 | 627 | 15.74 | 878 |
| 70-74 | 203 | 13.44 | 522 | 13.10 | 725 |
| 75+ | 142 | 9.40 | 276 | 6.93 | 418 |
| Education level |  |  |  |  |  |
| No education | 103 | 6.82 | 264 | 6.63 | 367 |
| Junior high school | 340 | 22.52 | 1589 | 39.88 | 1,929 |
| Some high school | 282 | 18.68 | 487 | 12.22 | 769 |
| High school graduate | 205 | 13.58 | 392 | 9.84 | 597 |
| Technical school, some college | 416 | 27.55 | 792 | 19.88 | 1,208 |
| College graduate | 164 | 10.86 | 460 | 11.55 | 624 |
| Race/Ethnicity |  |  |  |  |  |
| White | 1,118 | 74.04 | 2811 | 70.56 | 3,929 |
| Black | 51 | 3.38 | 68 | 1.71 | 119 |
| Hispanic | 17 | 1.13 | 37 | 0.93 | 54 |
| Asian and Pacific Islanders | 322 | 21.32 | 1,066 | 26.76 | 1,388 |
| Others | 2 | 0.13 | 2 | 0.05 | 4 |
| Region |  |  |  |  |  |
| Europe | 448 | 29.67 | 1,794 | 45.03 | 2,242 |
| North America | 740 | 49.01 | 1,125 | 28.24 | 1,865 |
| Asia & others | 322 | 21.32 | 1,065 | 26.73 | 1,387 |
| Study location |  |  |  |  |  |
| Milan (1984-1989) | 40 | 2.65 | 400 | 10.04 | 440 |
| Aviano | 51 | 3.38 | 180 | 4.52 | 231 |
| Italy multicenter | 131 | 8.68 | 637 | 15.99 | 768 |
| Swiss | 88 | 5.83 | 149 | 3.74 | 237 |
| Iowa | 195 | 12.91 | 308 | 7.73 | 503 |
| Rome | 70 | 4.64 | 162 | 4.07 | 232 |
| US multicenter | 352 | 23.31 | 431 | 10.82 | 783 |
| Japan1 | 128 | 8.48 | 621 | 15.59 | 749 |
| Japan2 | 194 | 12.85 | 444 | 11.14 | 638 |
| Buffalo | 193 | 12.78 | 386 | 9.69 | 579 |
| Milan (2006-2009) | 68 | 4.50 | 266 | 6.68 | 334 |
| Average amount of alcohol drinking (drinks/day) |  |  |  |  |  |
| 0 (Non-drinkers) | 546 | 36.16 | 1,882 | 47.24 | 2,428 |
| 0.1-0.9 | 382 | 25.30 | 1,058 | 26.56 | 1,440 |
| 1.0-2.9 | 263 | 17.42 | 694 | 17.42 | 957 |
| 3.0-4.9 | 144 | 9.54 | 119 | 2.99 | 263 |
| 5+ | 98 | 6.49 | 30 | 0.75 | 128 |
| Missing | 77 | 5.10 | 201 | 5.05 | 278 |
| Cumulative tobacco smoking (pack-years) |  |  |  |  |  |
| 0 (Never smoker) | 506 | 33.51 | 2536 | 63.65 | 3,042 |
| 0-10.0 | 95 | 6.29 | 391 | 9.81 | 486 |
| 10.1-20.0 | 110 | 7.28 | 246 | 6.17 | 356 |
| 20.1-30.0 | 154 | 10.20 | 188 | 4.72 | 342 |
| 30.1-40.0 | 127 | 8.41 | 132 | 3.31 | 259 |
| 40.1-50.0 | 136 | 9.01 | 78 | 1.96 | 214 |
| 50.0+ | 287 | 19.01 | 108 | 2.71 | 395 |
| Missing | 95 | 6.29 | 305 | 7.66 | 400 |
| Body Mass Index (BMI) (kg/m^2^) |  |  |  |  |  |
| Underweight (≤ 18.5) | 132 | 10.7 | 168 | 4.81 | 300 |
| Normal or healthy weight (18.5 to 24.9) | 771 | 62.5 | 2,058 | 58.9 | 2,829 |
| Overweight (25.0 – 29.9) | 250 | 20.3 | 934 | 26.7 | 1,184 |
| Obese (≥ 30) | 80 | 6.49 | 333 | 9.53 | 413 |

Alcohol drinking based on average of cumulative lifetime alcohol consumption, and assuming that one standardized drink contains 15.6 ml of pure ethanol.

Tobacco years combined cumulative lifetime use of cigarettes, cigars and pipes.

**Table S3. Associations between hormone and reproductive variables and all head and neck cancers in strata of body mass index (BMI, kg/m^2^), INHANCE Consortium**

|  | **Cases (E/Ē)** | **Controls (E/Ē)** | **OR (95% CI)** |
| --- | --- | --- | --- |
| Underweight (<18.5) |  |  |  |
| HRT use | 54/27 | 61/18 | 0.09 (0.005-1.57) |
| OC use | 6/29 | 8/42 | 39.07 (0.37-4120) |
| Ever given birth | 25/16 | 39/15 | 1.09 (0.10-11.25) |
|  |  |  |  |
| Normal weight (18.5-24) |  |  |  |
| HRT use | 348/174 | 814/308 | **0.43 (0.24-0.77)** |
| OC use | 58/192 | 186/656 | **0.53 (0.32-0.87)** |
| Ever given birth | 223/95 | 768/199 | **0.60 (0.41-0.89)** |
|  |  |  |  |
| Overweight (25-29) |  |  |  |
| HRT use | 75/72 | 256/208 | 0.48 (0.20-1.17) |
| OC use | 17/99 | 97/482 | 0.66 (0.32-1.39) |
| Ever given birth | 111/38 | 562/98 | 0.89 (0.47-1.71) |
|  |  |  |  |
| Obese (30+) |  |  |  |
| HRT use | 32/28 | 64/102 | 0.65 (0.16-2.61) |
| OC use | 7/27 | 41/187 | 1.35 (0.27-6.53) |
| Ever given birth | 35/10 | 231/48 | 1.16 (0.31-4.35) |

Models adjusted for study (and center for multicenter studies), age (<40, 40-44, 45-49, 50-54, 55-59, 60-64, 65-69, 70-74, 75+ years), education level (≤high school, > high school), amount of alcohol drinking (non-drinker, 0.1-0.9, 1.0-2.9, 3.0-4.9, and 5.0+ drinks/day), body mass index (normal weight, underweight, overweight, and obese), and cumulative tobacco smoking (never smoker, smoked 0-10.0, 10.1-20.0, 20.1-30.0, 30.1-40.0, 40.1-50.0, 50.0+ pack-years).

Abbreviations: E: exposed; Ē: un-exposed; HRT: hormone-replacement therapy; OC: oral contraceptives; OR: Odds ratios;
